# Different Approaches to Analyze Muscle Fat Replacement With Dixon MRI in Pompe Disease

**DOI:** 10.3389/fneur.2021.675781

**Published:** 2021-07-08

**Authors:** Alicia Alonso-Jiménez, Claudia Nuñez-Peralta, Paula Montesinos, Jorge Alonso-Pérez, Carme García, Elena Montiel, Izaskun Belmonte, Irene Pedrosa, Sonia Segovia, Jaume Llauger, Jordi Díaz-Manera

**Affiliations:** ^1^Neuromuscular Disorders Unit, Neurology Department, Departament de Medicina, Hospital de la Santa Creu I Sant Pau, Universitat Autònoma de Barcelona, Barcelona, Spain; ^2^Neuromuscular Reference Center, Neurology Department, University Hospital of Antwerp, Edegem, Belgium; ^3^Radiology Department, Hospital de la Santa Creu i Sant Pau, Universitat Autònoma de Barcelona, Barcelona, Spain; ^4^Philips Healthcare Iberia, Madrid, Spain; ^5^Biomedical Network Research Centre on Rare Diseases (CIBERER), Barcelona, Spain; ^6^Rehabilitation and Physiotherapy Department, Hospital de la Santa Creu i Sant Pau, Universitat Autònoma de Barcelona, Barcelona, Spain; ^7^John Walton Muscular Dystrophy Research Centre, Newcastle University, International Centre for Life, Newcastle upon Tyne, United Kingdom

**Keywords:** quantitative MRI, Dixon, Pompe, fat replacement, outcome measures

## Abstract

Quantitative MRI is an increasingly used method to monitor disease progression in muscular disorders due to its ability to measure changes in muscle fat content (reported as fat fraction) over a short period. Being able to objectively measure such changes is crucial for the development of new treatments in clinical trials. However, the analysis of the images involved continues to be a daunting task because of the time needed. Whether a more specific analysis selecting individual muscles or a global one analyzing the whole thigh or compartments could be a suitable alternative has only been marginally studied. In our study we compare three methods of analysis of 2-point-dixon images in a cohort of 34 patients with late onset Pompe disease followed over a period of one year. We measured fat fraction on MRIs obtained at baseline and at year 1, and we calculated the increment of fat fraction. We correlated the results obtained with the results of muscle function tests to investigate whether the three methods of analysis were equivalent or not. We observed significant differences between the three methods in the estimation of the fat fraction at both baseline and year 1, but no difference was found in the increment in fat fraction between baseline and year 1. When we correlated the fat fraction obtained with each method and the muscle function tests, we found a significant correlation with most tests in all three methods, although in most comparisons the highest correlation coefficient was found with the analysis of individual muscles. We conclude that the fastest strategy of analysis assessing compartments or the whole thigh could be reliable for certain cohorts of patients where the variable to study is the fat increment. In other sorts of studies, an individual muscle approach seems the most reliable technique.

## Introduction

In the past two decades, muscle MRI has been increasingly used for diagnosis of neuromuscular disorders. More recently, quantitative techniques such as Dixon or spectroscopy have been progressively implemented, as they provide the exact amount of fat present in skeletal muscles and can be used to follow up patients (1). In muscle spectroscopy, a region of interest (ROI) is drawn in the muscle when the patients are inside the MRI, and the analysis is only done in that specific region. In the case of Dixon, images are acquired and analyzed later on using specific software. Muscle fat content quantified using muscle MRI correlates with results of muscle function tests (2–4), and therefore, it is considered a good biomarker for neuromuscular diseases. Furthermore, quantitative muscle MRI is able to detect subtle changes in the amount of fat in muscles even before it impacts muscle function (3, 5). Quantitative muscle MRI is reproducible among different centers, and it is also harmless, as it does not use radiation. For its advantages, it has been proposed as a reliable outcome measure for natural history studies and clinical trials (6, 7). However, there is no standardized method to assess the fat content in skeletal muscle MRI acquired using Dixon sequence. Although several software solutions have been proposed to perform automatic or semiautomatic segmentation (8, 9), they are not widely accepted and used, and therefore, the analysis is continued manually, drawing ROIs in selected slices of the muscle. Analysis of Dixon images consumes a lot of time and requires a high degree of expertise and a profound knowledge of the anatomy of the muscles. Additionally, whether evaluating individual muscles is better than assessing compartments or even the whole limb has only been addressed in one study (10). Our aims for this study were as follows: (1) to check if different approaches to the quantification of fat replacement in thigh muscles of a cohort of patients with late-onset Pompe disease (LOPD) studied using Dixon quantitative muscle MRI showed differences at baseline and after 1-year follow-up, (2) to study whether there were significant differences in the increase of fat content after 1-year follow-up depending on the method used to analyze fat replacement, and (3) to identify which method of analysis correlated better with the results of the muscle function tests performed.

## Materials and Methods

### Cohort and Study Design

A total of 34 LOPD patients were included in this study. They were part of a larger prospective observational study following up LOPD patients registered in the webpage ClinicalTrials.gov with the identifier NCT01914536 (11). Inclusion criteria for the study were as follows: (1) diagnosis of LOPD based on recommendations recently proposed by the European Pompe Consortium (12) and reduced enzymatic activity in leukocytes, fibroblasts, or skeletal muscle and/or the presence of two mutations in the *GAA* gene; (2) no contraindications to MRI; and (3) willingness to complete all muscle function tests, respiratory assessment, and patient-reported outcome measures.

We collected the following epidemiological and clinical data: date of birth, gender, age at diagnosis, time of evolution of the disease, gene mutations, age at start of enzyme replacement therapy (ERT) treatment, current disease stage (ambulant or non-ambulant), and the need of (non)-invasive ventilation.

Patients were evaluated at baseline and 1 year after (±2 months). At each visit, muscle function tests and quantitative muscle MRI were performed. All patients provided written informed consent to participate in the study. The HSCSP ethics committee approved the study, and all participants signed an informed consent form. All study procedures were performed in accordance with Spanish regulations.

### MRI Acquisition and Analysis

Patients were examined in a 1.5T Ingenia MR system (Philips Healthcare, Best, the Netherlands) at HSCSP. Axial 3D fast field echo (FFE) Dixon sequence was acquired with the following parameters: repetition time/echo time (TR/TE) = 5.78/1.84 ms, flip angle = 15°, voxel size = 1 × 1 × 3 mm and field of view (FOV) 520 × 340 × 300 mm. We used the same position protocol for all patients: a supine position with the legs stretched out.

**Figure 1:**
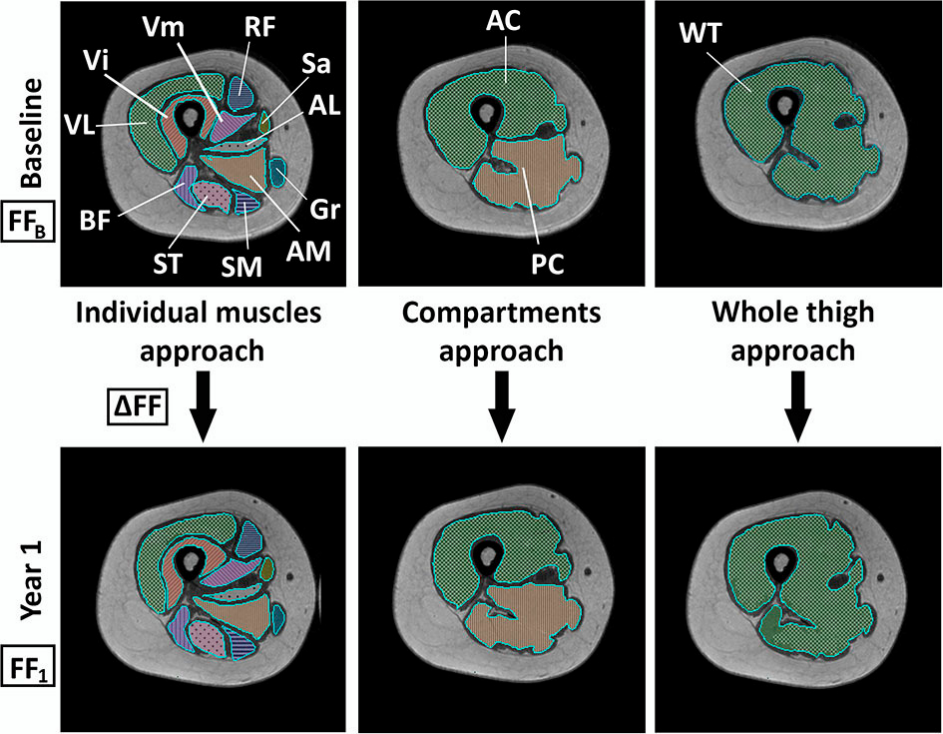
Schema of the three different analysis approaches at baseline visit and visit 1. VL, vastus lateralis; Vi, vastus intermedius; RF, rectus femoris; Vm, vastus medialis; Sa, sartorius; AL, adductor longus; Gr, gracilis; AM, adductor major; SM, semimembranosus; ST, semitendinosus; BF, biceps femoris; AC, anterior compartment; PC, posteromedial compartment; WT, whole thigh; FF_B_, fat fraction at baseline; ΔFF, fat fraction increment between baseline and visit 1; FF_1_, fat fraction at visit 1.

The percentage of fat in muscle or fat fraction (FF) was calculated with the PRIDE tool (Philips Research Image Development Environment) developed for this purpose. ROIs were manually drawn by one investigator (AA-J) on one slice in the right leg for each patient with three different approaches: firstly, by individual muscles (*rectus femoris, vastus intermedius, vastus lateralis, vastus medialis, adductor magnus, sartorius, gracilis, biceps femoris long head, semitendinosus*, and *semimembranosus*); secondly, by compartments (anterior compartment including the *rectus femoris, vastus intermedius, vastus lateralis, vastus medialis*, and *sartorius*; and posteromedial compartment including the *adductor magnus, gracilis, biceps femoris long head, semitendinosus*, and *semimembranosus*); and thirdly, including the whole thigh (Figure 1). To obtain the global thigh FF with the first two approaches, weighted averages were calculated by normalizing the FF by the area of the muscle/compartment (bigger muscles had higher weight). All ROIs were drawn in the same slice just below the *biceps femoris short head*. Neurovascular bundles and the femur were avoided. The analysis was performed in MRIs at baseline and after 1-year follow-up.

### Muscle Function Tests

All patients were studied by four physiotherapists (IB, CG, EM, and IP) with considerable experience in neuromuscular disorders at HSCSP in Barcelona. Each patient was examined by the same professional at baseline and after 1 year. The physiotherapists evaluated muscle function using the following tests: the 6-min walking test (6MWT), time to walk 10 m (10MWT), timed up-and-go test (TuGo), time to climb up (Tup4) and down four steps (Tdo4), the motor function measure-−20-item scale (MFM20), and the Muscle Research Council (MRC) scale in lower limbs. Patients also completed the self-reported questionnaire ActivLim (portmanteau of “ACTIVity LIMitations”). Global muscle function tests were selected instead of specific muscle tests to correlate with the muscle MRI because our analysis approach measured the global FF of the thigh. We obtained forced vital capacity, both seated and lying down, using the Carefusion Microlab ML 3500 MK8 spirometer (Care Fusion, Yorba Linda, CA, USA).

### Statistical Analysis

We used the Shapiro–Wilk-test to check if our variables followed a normal distribution. As they did not, we used non-parametric statistic tests for the analysis. We used the Friedman test to compare the FF and the increment of FF obtained with the three methods of analysis as well as the time necessary to perform the analysis. In case of a global significant effect, pairwise comparisons are performed with Wilcoxon signed-rank tests. We used the paired Wilcoxon signed-rank test to investigate if there were significant changes in the FF obtained between baseline and visit 1. To investigate the correlations between the muscle function tests and FF, we used Spearman's correlation. We considered that correlations were good if p was lower than 0.05 and the correlation coefficient (rho) was 0.65 or higher. To investigate whether the differences between the correlations with the three different approaches were significant or not, we used the web utility provided by quantpsy.org (13). Graphs and statistical analysis were performed using IBM® SPSS® Statistics Version 21.

## Results

### Clinical Features of Patients

We included a total of 34 LOPD patients in this study of whom 19 were women (55.9%) and 15 were men (44.1%). Clinical data of the patients are summarized in Table 1. The mean age at first visit was 45.6 years (SD: 14.22 years), and the mean time from onset of symptoms was 14.03 years (SD = 9.47 years). Two patients were homozygous, one for the mutation c.2173C>T and the other one for the mutation c.−113+2T>A. The rest were compound heterozygous. Most patients carried the classical inversion IVS1-13T>G. Seven patients had only hyperCKemia with normal strength and were considered asymptomatic. Among the 27 symptomatic patients, 14 had exclusively limb muscle weakness, whereas 13 had respiratory and limb muscle involvement. A total of 32 patients were ambulant (two of whom used a walker), and only two needed a manual wheelchair. A total of 23 patients were treated with ERT at the time of the baseline visit.

**Table 1:** Clinical characteristics of the patients.

**Patient**	**Sex**	**Age at study (years)**	**Wheelchair dependent**	**Phenotype**	**GAA mutation 1**	**GAA mutation 2**	**Age at ERT**	**Respiratory support**
1	F	50	No	Muscular	IVS1-13T>G	c.1076-1G>C	47	No
2	F	48	Yes	Muscular + respiratory	IVS1-13T>G	c.2173C>T	37	Yes
3	F	26	No	HyperCKemia	IVS1-13T>G	c.1889-1G>A	-	No
4	F	63	No	Muscular	IVS1-13T>G	c.2600_2604delTGCTGinsA	59	No
5	F	47	No	Muscular	IVS1-13T>G	c.15323c>A	42	No
6	F	51	No	Muscular	IVS1-13T>G	c.236_246del	47	No
7	M	66	No	Muscular + respiratory	IVS1-13T>G	c.1933G>T	-	No
8	F	59	No	Muscular	IVS1-13T>G	c.1637A>G	52	No
9	F	55	No	Muscular	IVS1-13T>G	c.2173C>T	48	No
10	M	42	No	Muscular + respiratory	IVS1-13T>G	c.573C>A	39	Yes
11	F	31	Yes	Muscular + respiratory	IVS1-13T>G	c.1637A>G	24	Yes
12	F	47	No	Muscular	IVS1-13T>G	c.1192dupC	39	No
13	M	47	No	Muscular + respiratory	c.2173C>T	c.2173C>T	45	Yes
14	M	51	No	Muscular + respiratory	IVS1-13T>G	c.1657C>T	45	Yes
15	F	51	No	Muscular + respiratory	IVS1-13T>G	c.1657C>T	46	Yes
16	M	24	No	HyperCKemia	IVS1-13T>G	c.1802C>T	-	No
17	M	51	No	HyperCKemia	c.271G>A	c.2510G>A	-	No
18	M	14	No	HyperCKemia	IVS1-13T>G	c.281_282delCT	-	No
19	F	67	No	Muscular	c.1781G>A	c.1194mas5G>A	64	No
20	F	35	No	Muscular	IVS1-13T>G	c.1A>T	29	No
21	F	40	No	Muscular + respiratory	IVS1-13T>G	c.1889-1G>A	-	Yes
22	F	53	No	Muscular	c.1781G>A	c.1194+5G>A	45	No
23	M	66	No	Muscular + respiratory	IVS1-13T>G	c.2481+102_2646+31del	57	Yes
24	M	8	No	HyperCKemia	IVS1-13T>G	c.1889-1G>A	-	No
25	F	57	No	Muscular + respiratory	IVS1-13T>G	c.1447G>T	55	Yes
26	M	46	No	Muscular + respiratory	IVS1-13T>G	c.15323c>A	43	Yes
27	M	51	No	Muscular + respiratory	IVS1-13T>G	c.1933G>T	51	Yes
28	M	51	No	Muscular	IVS1-13T>G	c.1933G>T	-	No
29	M	43	No	Muscular	IVS1-13T>G	c.1408a1410delC>T	43	No
30	F	54	No	Muscular	c.-113+2T>A	c.-113+2T>A	48	No
31	M	51	No	HyperCKemia	IVS1-13T>G	c.1637A>G	-	Yes
32	M	43	No	Muscular + respiratory	IVS1-13T>G	c.655G>A	-	No
33	F	20	No	HyperCKemia	IVS1-13T>G	c.1551+1G>A	-	No
34	F	41	No	Muscular	IVS1-13T>G	c.1655T>C	40	No

*ERT, enzyme replacement therapy*.

### Differences in Fat Fraction Among the Three Methods of Analysis at Baseline and Visit 1

At baseline, the mean FF of the thigh calculated by analyzing individual muscles and performing then a weighted average (analysis by individual muscles) was 38.98% (SD: 23.58). The FF obtained by analyzing anterior and posteromedial compartment and then doing a weighted average (analysis by compartments) was 39.08 (SD: 22.45). The FF obtained by the whole-thigh approach was 42.48 (SD: 23.55). Friedman's-test showed that the differences in the average FF among the three methods at baseline were significant (*p* ≤ 0.001). Pairwise comparisons showed a significant difference between the analysis by individual muscles and the analysis of the whole thigh, and between the analysis by compartments and the analysis of the whole thigh, but not between analysis by individual muscles and compartments (respectively, *p* < 0.001, *p* < 0.001, and *p* = 0.825).

**Figure 2:**
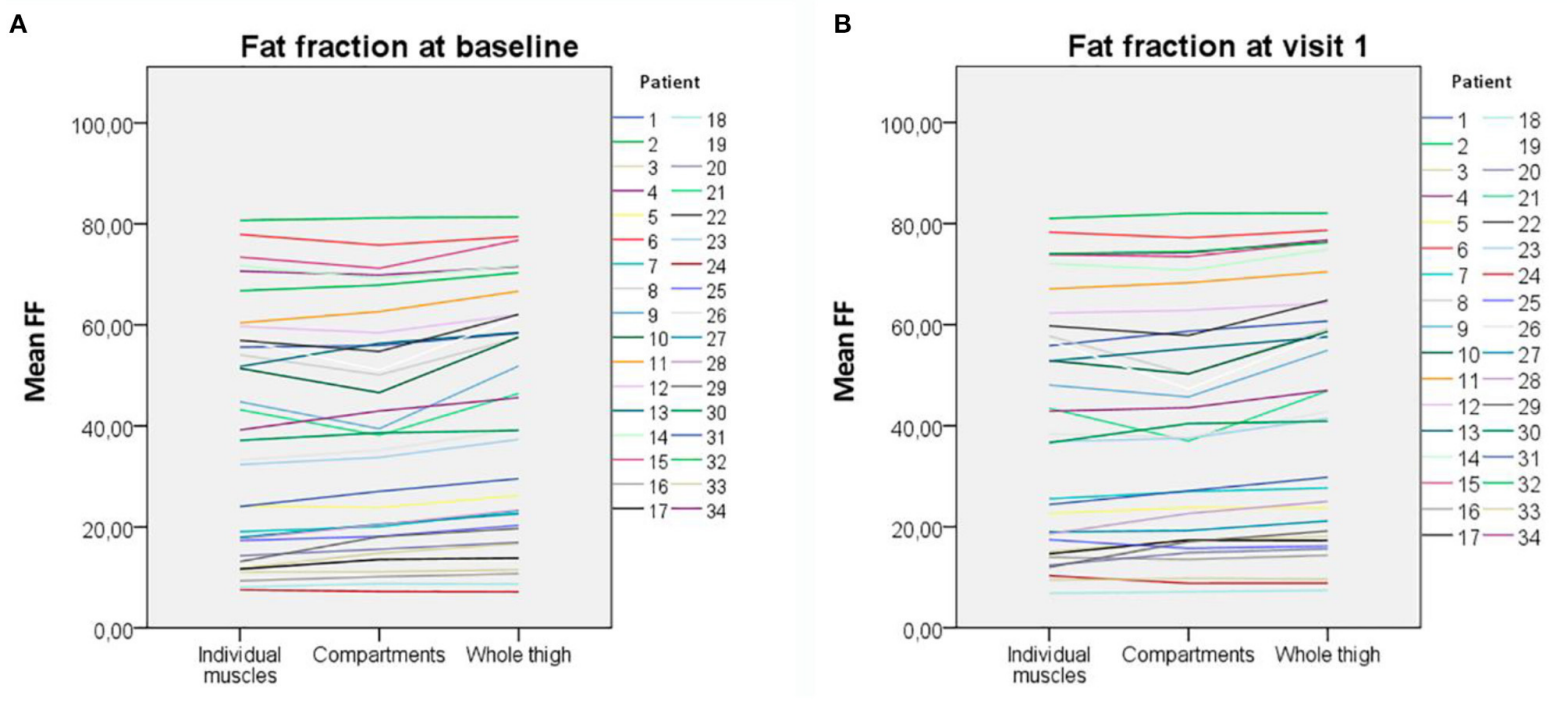
Fat fraction obtained with the three methods of analysis for each patient. **(A)** At baseline. **(B)** At visit 1. The variability is higher in patients with middling fat replacement and lower in both patients with very high and very low fat replacement.

At visit 1, the mean FF in the individual muscles analysis was 40.81 (SD: 24.05), by compartments was 40.79 (SD: 23.27), and by the whole thigh was 43.76 (SD: 24.34). These differences were also significant between the analysis by individual muscles and the analysis of the whole thigh, and between the analysis by compartments and the analysis of the whole thigh (*p* < 0.001) and not significant between analyses by individual muscles and compartments (respectively, *p* < 0.001, *p* < 0.001, and *p* = 0.996). Figure 2 shows the FF obtained with the three methods for each patient of the study.

We measured the time needed to draw the ROIs by each approach in a subset of 15 patients. The mean time per patient to analyze the MRI by individual muscles in both visits was 330.67 s (SD: 22.71), by compartments 179.09 s (SD 12.87), and by the whole thigh 114.7 s (SD 12.97). These differences were significant for the three approaches (Friedman-test, *p* < 0.001).

### Fat Fraction Changes After 1 Year of Follow-Up and Differences Among the Three Methods of Analysis

**Figure 3:**
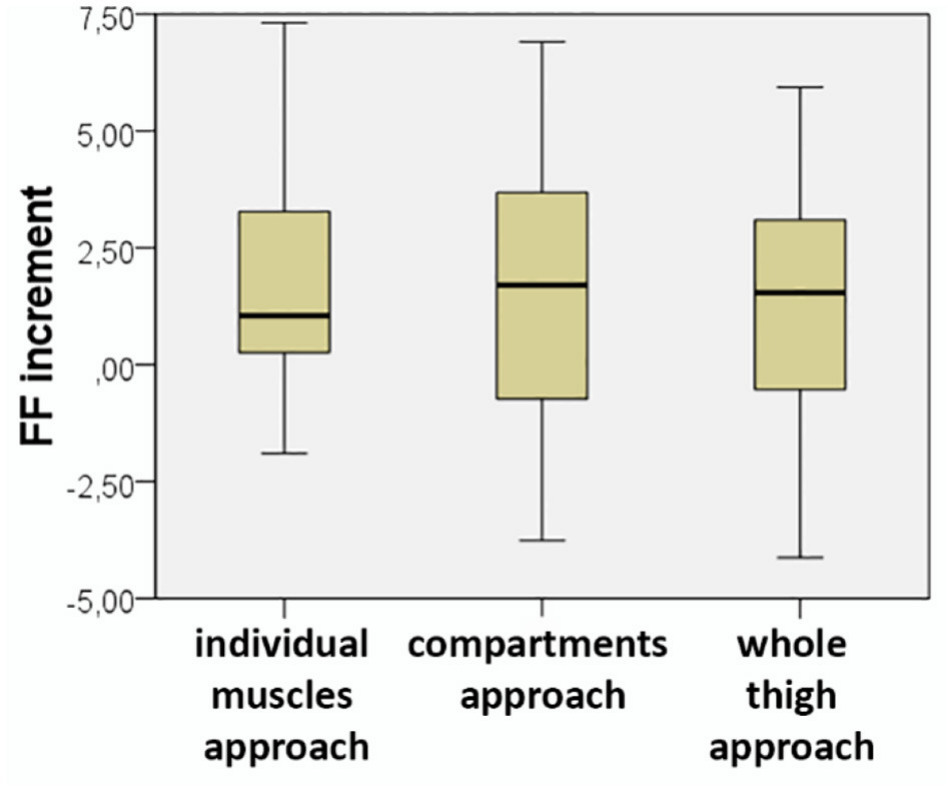
Increment in fat fraction obtained with the three methods of analysis. There were no significant differences among them (Friedman *p* = 0.2).

The mean increment in FF, calculated by subtracting the FF at baseline visit from the FF at visit 1, was 1.83% (SD: 2.48) for the individual muscles analysis, 1.71% (SD: 2.68) for the compartments analysis, and 1.28% (SD: 2.44) for the whole-thigh approach. A Wilcoxon signed-rank test showed that this difference in FF between baseline and visit 1 was significant for the three approaches (by individual muscles *Z* = −3.462, *p* = 0.001; by compartments *Z* = −3.171, *p* = 0.002; and by whole thigh *Z* = −2.727, *p* = 0.006). In fact, the mean FF increment was not statistical significantly different among the three methods of analysis (Figure 3).

### Correlation With Muscle Function Tests

**Figure 4:**
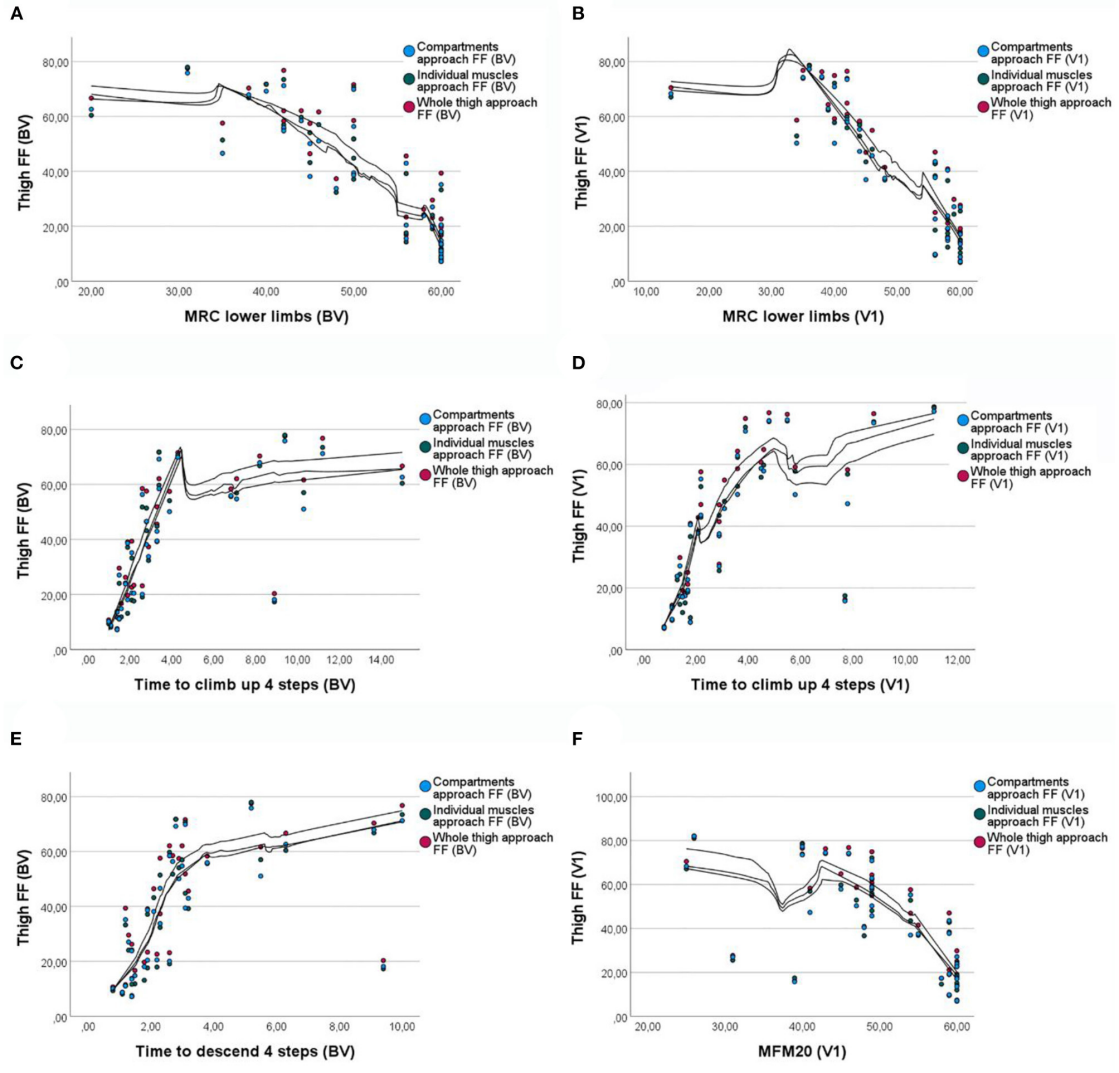
Correlations between fat fraction and muscle function tests with the three approaches. **(A)** Correlation between MRC in lower limbs and FF at baseline. **(B)** Correlation between MRC in lower limbs and FF at visit 1. **(C)** Correlation between the time to climb four steps test and FF at baseline. **(D)** Correlation between the time to climb four steps test and FF at visit 1. **(E)** Correlation between the time to descend four steps test and the FF at baseline. **(F)** Correlation between the MFM20 and FF at visit 1. FF, fat fraction; BV, baseline visit; V1, visit 1; MRC, Muscle Research Council scale; MFM20, motor function measure−20-item scale.

After our observation that there were statistically significant differences between the three analysis strategies, we wondered whether these differences were clinically significant or not. Correlations between FF and muscle function tests have been previously described in LOPD patients (2). We confirmed that there was a correlation between FF and some muscle function tests and patient-reported outcome measures, which we considered could be affected by fat replacement in the thigh. 10MWT, MRC in lower limbs, MFM20, Tup4, and Tdo4 showed strong correlations with the FF both at baseline and at visit 1 with the three methods of analysis (Figure 4). 6MWT and TuGo showed good correlation at visit 1 but not at the baseline. The correlation coefficient was slightly higher in the approach by individual muscles for all the correlations except MRC in lower limbs at visit 1, where the coefficient was slightly higher by whole-thigh analysis (Table 2). We investigated whether these differences among the correlation coefficients were significant or not and found that in eight out of the 13 comparisons with strong correlation coefficients, they were indeed significantly different.

**Table 2:** Correlations between fat fraction and muscle function tests with the three methods of analysis.

	**Baseline**						**Significant difference?**	**Visit 1**						**Significant difference?**
**Type of analysis**	**By muscles**		**By compartments**		**By whole thigh**			**By muscles**		**By compartments**		**By whole thigh**		
**Test**	***p***	**Spearman's rho**	***p***	**Spearman's rho**	***p***	**Spearman's rho**		***p***	**Spearman's rho**	***p***	**Spearman's rho**	***p***	**Spearman's rho**	
10MWT	0.00	**0.8**	0.00	0.769	0.00	0.780	Yes	0.00	**0.796**	0.00	0.738	0.00	0.753	Yes
MRC LL	0.00	**−0.885**	0.00	−0.866	0.00	−0.881	No	0.00	−0.891	0.00	−0.880	0.00	**−0.892**	No
MFM20	0.00	**−0.681**	0.00	−0.656	0.00	−0.661	No	0.00	**−0.725**	0.00	−0.681	0.00	−0.682	Yes
TuGo	0.039	0.373	0.056		0.054		No	0.00	**0.791**	0.00	0.729	0.00	0.744	Yes
Tup4	0.00	**0.846**	0.00	0.822	0.00	0.835	Yes	0.00	**0.840**	0.00	0.777	0.00	0.805	Yes
Tdo4	0.00	**0.764**	0.00	0.756	0.00	0.749	No	0.00	**0.754**	0.00	0.694	0.00	0.716	Yes
6MWT	0.00	**−0.658**	0.00	−0.634	0.00	−0.641	No	0.00	**−0.773**	0.00	−0.724	0.00	−0.734	Yes
Activlim	0.00	−0.607	0.00	**−0.619**	0.00	−0.604	No	0.00	−0.494	0.00	**−0.497**	0.01	−0.469	No

*The highest correlation coefficients have been remarked in bold for each comparison.*

*10MWT, time to walk 10 m; MRC LL, Muscle Research Council Scale in lower limbs; MFM20, motor function measure−20-item scale; TuGo, timed up-and-go test; Tup4, time to climb up four steps; Tdo4, time to climb down four steps; 6MWT, 6-min walking test; Activlim, Activity Limitations questionnaire*.

## Discussion

In the present paper, we compared three methods of analysis of FF in the thigh in a cohort of LOPD patients. There is no standardized method to perform this analysis to date. In most published studies, analysis is performed by manually drawing ROIs in individual muscles in several slices of the thighs (between three and five in most of the studies), which is highly time-consuming (3, 5, 14, 15). Drawing ROIs over the whole thigh or over the anterior and posteromedial compartments could reduce the time of analysis. Whether these different approaches are clinically or statistically significant has only recently been approached by one group (10). We have shown here that there is a significant difference in the time used between the three strategies: compared with the analysis by individual muscles, it was 45.84% faster when performed by compartments and 65.31% by the whole-thigh approach. In studies involving a large number of MRIs, this difference can mean a lot of hours.

In their study, Reyngoudt et al. (10) performed a comparative analysis of different segmentation approaches in 102 patients with different muscle disorders. They conclude that the increment in FF calculated by global analysis of the whole thigh is reliable in most muscle disorders. Our study supports these findings, as we did not find a significant difference in the increment of FF between the three approaches. The added value of our study is that we compared the three methods of analysis at baseline and visit 1, and we correlated them with muscle function tests. The comparisons between the average FF using the three methods showed statistical differences at both baseline and visit 1. The pairwise comparison showed that the differences were found between the analysis by individual muscles and by the whole thigh, and by compartments and the whole thigh, but not between individual muscles and compartments. Interestingly, the mean FF was higher for the whole-thigh analysis in both visits. We hypothesized that this higher content in fat could be due to intermuscular fat, which can be affected by other variables besides the muscle disease such as the index body mass (16). This could explain the statistically significant difference with the other two approaches.

However, when we looked into each patient in detail (Figure 2), we observed that the variability between the three methods of analysis is higher for patients with intermediate amount of fat replacement (20–60%), whereas patients with low (<20%) or very high (>60%) fat content tend to be more homogenous. This finding might indicate that in studies with cohorts of patients with very low fat replacement (such as asymptomatic and pauci-symptomatic patients), a more general approach with analysis by compartments of the whole thigh may be appropriated.

We wondered whether the difference in FF between the three methods could also be clinically significant. In order to clarify it, we investigated the correlations between the FF obtained by each of the methods and muscle function tests and the ActivLim. We found strong correlation coefficients (rho ≥ 0.65) between FF and 10MWT, MRC in lower limbs, MFM20, Tup4, and Tdo4 in both visits and 6MWT in visit 1. Although the correlation was found for the three methods of analysis, the coefficient was higher for the individual muscles approach in all but one of the comparisons with a high coefficient. Furthermore, the differences between the correlation coefficients were significant for the majority of functional tests, pointing out that the differences between the different approaches of analysis are not only statistically significant but also clinically relevant.

Our study suggests that, in general, Dixon MRI analysis seems to correlate better with muscle function tests if the manual input is executed by drawing ROIs in individual muscles and obtaining later the global thigh FF with weighted averages. This approach has also the advantage of providing information about individual muscles. Myopathies and muscular dystrophies are characterized by a heterogeneous muscle fat replacement, affecting different muscles in different diseases. Furthermore, the rate of muscle degeneration is not the same for all the muscles: for example, in Pompe disease, the *adductor magnus* is early affected, whereas the *sartorius* or *gracilis* is spared until late stages of the disease. Therefore, the former can be a good muscle to monitor progression at the beginning of the disease, while the latter could be used in late stages.

However, in specific situations, less time-consuming approaches such as drawing ROIs by compartments or in the whole thigh might be used with good reliability, for instance, in cohorts of patients with very low muscle fat replacement. These approaches could also be appropriated if we are interested in quantifying the increment in fat replacement between several visits.

Our study has two main limitations. Firstly, we have studied only patients with Pompe disease; therefore, studies in other type of muscle disorders and muscular dystrophies could show different results. Secondly, we have performed the study only in one slice in the thighs. Analysis of more slices would probably provide a better approximation to the real amount of fat in muscle. Despite its limitations, we think that our study is useful because it approaches both the statistical and clinically significant differences obtained with three different analysis strategies.

In conclusion, the method of analysis used to quantify the fat replacement in muscle using Dixon sequences should be carefully chosen, taking into account the goal of the study as well as the characteristics of the patients. In transversal studies where the variable to study is the FF, or in subjects with moderate fat replacement, an analysis by individual muscles seems to be more accurate. On the other hand, in longitudinal studies where the variable of interest is the increment in FF, or in cohorts of patients with little fat replacement, a global approach by analyzing compartments or the whole muscle could be more efficient.

## Data Availability Statement

The raw data supporting the conclusions of this article will be made available by the authors under request, without undue reservation.

## Ethics Statement

The studies involving human participants were reviewed and approved by Hospital de la Santa Creu i Sant Pau ethics committee. Written informed consent to participate in this study was provided by the participants' legal guardian/next of kin.

## Author Contributions

AA-J designed the protocol, visited the patients, analyzed muscle MRIs, and wrote the paper. CN-P collaborated with the design of the protocol, offered technical support for neuroimaging, and contributed to the final version of the paper. JA-P visited the patients and managed the database. JL offered technical support for neuroimaging and reviewed the paper. SS coordinated the study, managed the database, and reviewed the paper. IB, IP, CG, and EM visited the patients, acquired motor function data, and contributed to the final version of the manuscript with their comments. PM offered technical assistance with the MRIs, verified the quality of the acquired images and contributed to the final version of the manuscript. JD-M designed the protocol, visited the patients, wrote the paper, and obtained funding for the study. All authors contributed to the article and approved the submitted version.

## Conflict of Interest

PM is employed by the company Philips Healthcare Iberia. The remaining authors declare that the research was conducted in the absence of any commercial or financial relationships that could be construed as a potential conflict of interest.
